# MCM-UNet++: A Hybrid Soft Computing Framework for Multi-Scale Polyp Segmentation via Enhanced Global Context and Adaptive Feature Fusion

**DOI:** 10.3390/s26113380

**Published:** 2026-05-26

**Authors:** Jinmei Li, Ming Zhao, Quan Du, Song Lu, Shenglung Peng

**Affiliations:** 1 School of Computer Science, Yangtze University, Jingzhou 434025, China; 2024720785@yangtzeu.edu.cn (J.L.); 2024710726@yangtzeu.edu.cn (Q.D.); 2 School of Cyber Science and Engineering, Wuxi University, Wuxi 214105, China; lusong@cwxu.edu.cn; 3 Department of Creative Technologies and Product Design, National Taipei University of Business, Taipei 10051, Taiwan; slpeng@ntub.edu.tw

**Keywords:** polyp segmentation, soft computing, Multi-Axis Transformer, adaptive feature fusion, multi-objective loss, U-Net++

## Abstract

Colonoscopy polyp segmentation is important for colorectal cancer screening, yet it remains challenging because polyps exhibit large morphological variation, weak lesion–background contrast, blurred boundaries, and severe foreground–background imbalance. To address these issues, this paper presents MCM-UNet++, a hybrid U-Net++-based segmentation framework that combines three targeted enhancements. First, a Multi-Axis Transformer Block (MATransformerBlock) is incorporated into convolutional feature blocks to model long-range horizontal and vertical dependencies with lower complexity than dense global self-attention. Second, a Cross-Channel Mixing (CCM) module is used in nested skip fusion paths to recalibrate the channel and spatial responses and reduce redundant feature transmissions. Third, a Multi-Objective Adaptive Loss (MOALoss) combines focal, Dice, and boundary-aware terms with learnable weights to improve supervision for small regions and ambiguous boundaries. Experiments on four public polyp segmentation datasets (Kvasir-SEG, CVC-ClinicDB, CVC-ColonDB, and ETIS-Larib) show competitive performance against the selected baseline methods, with Dice/IoU scores of 0.9563/0.9278 on Kvasir-SEG and 0.8593/0.7896 on CVC-ColonDB. These results indicate that the proposed components can improve benchmark-level polyp segmentation performance, while broader validation is still required before clinical deployment.

## 1. Introduction

Colorectal cancer (CRC) stands as a leading cause of cancer-related mortality worldwide, and the early detection and segmentation of colonic polyps through colonoscopy represent a critical procedure for its prevention and diagnosis [1]. The objective of automated polyp segmentation is to precisely delineate polyp regions from colonoscopy images, thereby providing clinicians with indispensable support for localization and assessment. However, the practical deployment of such systems is severely hampered by several inherent challenges: the high morphological heterogeneity of polyps, ranging from flat to pedunculated structures [2,3]; the low contrast between lesions and surrounding mucosa, particularly under conditions of inflammation or bleeding, leading to blurred boundaries [4]; and a pronounced foreground–background class imbalance.

Convolutional Neural Networks (CNNs), particularly U-Net and its variants, such as U-Net++ [5] and ResUNet++ [6], have established themselves as the de facto standard in medical image segmentation. By leveraging symmetric encoder–decoder architectures with skip connections, these models have achieved commendable performances, with average Dice scores around 85% on benchmark datasets. Despite their success, their reliance on stacked convolutional operations for receptive field expansion inherently limits their capacity to model long-range spatial dependencies—a crucial aspect for identifying the global context of irregularly shaped polyps. Furthermore, the standard skip connection mechanism, while fusing multi-level features, often introduces redundancy and fails to preserve the high-frequency details that are essential for accurate boundary delineation, especially for diminutive polyps. Lastly, the local inductive bias of CNNs can lead to significant performance degradation when faced with distribution shifts induced by different endoscopic imaging devices. This limitation is also reflected in context aggregation and attention-based segmentation studies [7,8].

The advent of Transformer architectures [9,10], pioneered in natural language processing, has introduced a paradigm shift in computer vision. The Vision Transformer (ViT) [10] and its successors (e.g., Swin Transformer [11], TransUNet [12]) demonstrate remarkable prowess in capturing global contextual information through self-attention mechanisms, outperforming CNNs in various tasks, including colonoscopy image classification. However, a direct transplantation of Transformers for dense, pixel-level segmentation encounters two formidable obstacles. First, the self-attention mechanism exhibits quadratic computational complexity with respect to image resolution, rendering it computationally prohibitive for high-resolution medical images. Second, standard multi-head self-attention, while effective for global modeling, can be less sensitive to fine-grained local textures, potentially resulting in boundary artifacts that are detrimental to precise segmentation.

This dichotomy between the local feature excellence of CNNs and the global context mastery of Transformers underscores a fundamental challenge in medical image analysis. It suggests that purely convolutional or purely Transformer-based approaches may be approaching their performance ceilings. This realization has catalyzed the exploration of hybrid architectures, which seek a synergistic integration of these two paradigms; while initial hybrid models have shown promise, the depth of this integration—moving beyond simple replacement of encoder components—remains underexplored. The core research question persists: how can we most effectively intertwine local feature extraction with global semantic modeling to navigate the specific challenges of polyp segmentation?

Recent network studies provide useful but still-incomplete solutions to this question. U-Net variants improve encoder–decoder fusion by adding dense skip paths, recurrent blocks, deformable convolutions, or lightweight modules [5,13,14,15,16]. Transformer-based and hybrid models, such as Swin Transformer and DS-TransUNet, strengthen long-range dependency modeling and multi-scale contextual interactions [11,12,17]. However, many of these methods either increase computational cost, rely on direct feature concatenation in skip paths, or use fixed loss weighting. For polyp segmentation, these issues are particularly relevant because small lesions, unclear boundaries, and background textures require both selective feature fusion and boundary-sensitive optimization.

In response, this paper proposes MCM-UNet++, a U-Net++-based segmentation network that integrates axial global context modeling, adaptive skip-path feature selection, and multi-objective loss balancing. Rather than claiming a completely independent segmentation paradigm, the proposed method is positioned as a targeted enhancement of U-Net++ for polyp segmentation. The contributions of this work are threefold:

- We introduce a Multi-Axis Transformer Block (MATransformerBlock) into convolutional feature blocks. By decomposing attention into horizontal and vertical pathways, the module captures long-range dependencies while avoiding the full quadratic cost of dense self-attention.
- We design a Cross-Channel Mixing (CCM) module for nested skip fusion paths. Unlike direct concatenation or single attention gates, CCM combines channel recalibration and spatial filtering to suppress redundant responses and emphasize task-relevant boundary features.
- We formulate a Multi-Objective Adaptive Loss (MOALoss) that combines focal, Dice, and boundary-aware objectives with learnable weights, thereby providing more balanced supervision for hard foreground pixels, region overlap, and boundary refinement.

Experiments on four public benchmarks—Kvasir-SEG, CVC-ClinicDB, CVC-ColonDB, and ETIS-Larib—show that MCM-UNet++ achieves competitive Dice and IoU values among the selected baselines. The ablation study further suggests that CCM, MATransformerBlock, and MOALoss provide complementary improvements.

## 2. Materials and Experimental Protocol

This section describes the datasets, preprocessing, evaluation metrics, and training protocol before presenting the network methodology. This organization separates the experimental design from the model description.

### 2.1. Datasets and Data Splits

We evaluated MCM-UNet++ on four public polyp segmentation datasets. Kvasir-SEG contains 1000 endoscopic images with pixel-level polyp masks. CVC-ClinicDB contains 612 images extracted from 29 colonoscopy videos. CVC-ColonDB contains 380 images with a native resolution of 288×384 pixels. ETIS-Larib contains 196 images from 29 short endoscopic sequences and includes challenging cases with blurred boundaries and variable textures.

All experiments in this study used the same fixed random partitioning protocol: 80% of the images were used for training and 20% for validation, with the data split fixed by random_state=41. The Kvasir-SEG 880:120 split is a commonly used alternative protocol, but it is not mixed with the present experimental results. Table 1 summarizes the split protocol.

### 2.2. Preprocessing and Data Augmentation

The training set was processed with an Albumentations-based augmentation pipeline, including random 90° rotation, horizontal and vertical flipping, color perturbation, resizing, cropping to the configured input size, and normalization. The validation images were resized and normalized without stochastic augmentation. Images and masks were transformed synchronously to preserve pixel-level alignment. In the reported runs, Kvasir-SEG, CVC-ClinicDB, and CVC-ColonDB used 96×96 inputs, whereas ETIS-Larib used 128×128 inputs, as listed in Table 2. These relatively small input sizes were selected to keep the full nested U-Net++ structure, MATransformerBlock, and CCM modules trainable on the available NVIDIA RTX 3060 Laptop GPU. We recognize that downsampling may remove fine boundary details; therefore, higher-resolution training and resolution ablation are discussed as future work in Section 4.4.

### 2.3. Evaluation Metrics

To provide a more complete evaluation of binary polyp segmentation, we used overlap, pixel classification, probability error, and boundary distance metrics. The main overlap metrics are Dice and Intersection over Union (IoU). Dice is sensitive to small target overlap, whereas IoU penalizes both false-positive and false-negative regions more strictly. They are defined as

(1) $$\mathrm{Dice}\left(P,G\right)=\frac{2\sum_{i}p_{i}g_{i}}{\sum_{i}p_{i}+\sum_{i}g_{i}},$$

(2) $$\mathrm{IoU}\left(P,G\right)=\frac{\sum_{i}p_{i}g_{i}}{\sum_{i}p_{i}+\sum_{i}g_{i}-\sum_{i}p_{i}g_{i}}.$$

Here, *P* and *G* denote the predicted and ground-truth masks, and $p_{i},g_{i}\in \left\{0,1\right\}$ denote the binary pixel values at location *i*. Let TP, TN, FP, and FN denote true-positive, true-negative, false-positive, and false-negative pixels after thresholding the predicted probability map at 0.5. Precision, Recall/Sensitivity, Specificity, and Accuracy are computed as follows:

(3) $$\begin{array}{cccc} \mathrm{Precision} & =\frac{\mathrm{TP}}{\mathrm{TP}+\mathrm{FP}}, & \mathrm{Recall} & =\frac{\mathrm{TP}}{\mathrm{TP}+\mathrm{FN}},\\ \mathrm{Specificity} & =\frac{\mathrm{TN}}{\mathrm{TN}+\mathrm{FP}}, & \mathrm{Accuracy} & =\frac{\mathrm{TP}+\mathrm{TN}}{\mathrm{TP}+\mathrm{TN}+\mathrm{FP}+\mathrm{FN}}. \end{array}$$

Precision measures false-positive control, Recall/Sensitivity measures missed-polyp risk, Specificity measures background rejection, and Accuracy summarizes pixel-level correctness. We also report mean absolute error (MAE), which measures the average deviation between the predicted probability map $\hat{P}$ and the binary ground-truth mask *G*:

(4) $$\mathrm{MAE}=\frac{1}{N}\sum_{i=1}^{N}\left|{\hat{p}}_{i}-g_{i}\right|.$$

Boundary Accuracy is evaluated using the 95th percentile Hausdorff distance (HD95):

(5) $$\mathrm{HD}95\left(P,G\right)=\max\left\{{\mathrm{percentile}}_{95}\left(d(\partial P,\partial G)\right),{\mathrm{percentile}}_{95}\left(d(\partial G,\partial P)\right)\right\},$$

where ∂P and ∂G denote the predicted and ground-truth boundaries, and d(·,·) denotes the nearest-boundary Euclidean distance. Lower MAE and HD95 values indicate better probability calibration and boundary localization, respectively.

### 2.4. Implementation and Training Details

All experiments were implemented in PyTorch 2.2.2 with CUDA 11.8 on Windows 10, using an NVIDIA GeForce RTX 3060 Laptop GPU and an AMD Ryzen 7 5800H CPU. The reported runs used one binary output channel and dataset-specific epoch, batch size, input resolution, and momentum settings, which are listed in Table 2. The optimizer was SGD with a weight decay of $1\times 10^{-4}$, no Nesterov acceleration, and an initial learning rate of $1\times 10^{-3}$. A cosine annealing learning-rate scheduler was used with a minimum learning rate of $1\times 10^{-5}$, and early stopping was disabled. Data loading used zero workers in the reported configuration for cross-platform stability. The reported values correspond to the best validation checkpoint under the fixed split with random_state=41; repeated independent runs and standard deviations are listed as a limitation and future extension because they were not available in the current experimental records.

## 3. Methodology

This section focuses on the proposed method. MCM-UNet++ is built on the nested U-Net++ topology and modifies three parts of the original framework: the convolutional feature blocks, the nested skip fusion paths, and the training objective.

**Figure 1:**
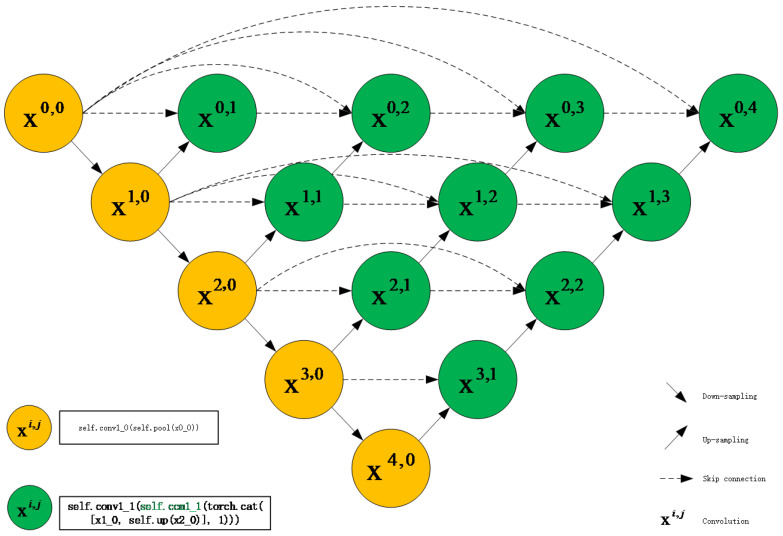
Overall framework of MCM-UNet++. The caption identifies the encoder, MATransformerBlock- enhanced feature blocks, CCM-based nested skip fusion, decoder, prediction head, and MOALoss supervision.

Figure 1 illustrates the overall architecture. Given an input colonoscopy image, the encoder extracts multi-level features with channel dimensions {32,64,128,256,512}. Each feature block contains convolutional operations followed by MATransformerBlock-based global context modeling. In the decoder, nested skip fusion nodes concatenate encoder and decoder features and pass them through CCM before subsequent convolutional refinement. The final 1×1 convolution generates the binary polyp probability map, and MOALoss supervises the prediction by jointly considering hard pixels, region overlap, and boundary regions. The main architectural configuration is summarized in Table 3.

For MATransformerBlock, the number of attention heads was set to 4 at each level, so the head dimension was C/4. The MLP expansion ratio was 4, i.e., the hidden dimension was 4C, and no dropout was used in the reported implementation. CCM was inserted at all ten nested decoder fusion nodes of U-Net++, with a channel attention reduction ratio of 2 and a 3×3 spatial attention convolution kernel.

### 3.1. MATransformerBlock: Efficient Multi-Axis Global Context

MATransformerBlock is introduced to compensate for the limited receptive field of convolutional operations while avoiding the high cost of dense self-attention. In this paper, *B* denotes batch size, *C* denotes the number of channels, and H×W consistently denotes the spatial resolution of the current feature map. For the *l*-th network level, the spatial size can be written as $H_{l}\times W_{l}$.

**Figure 2:**
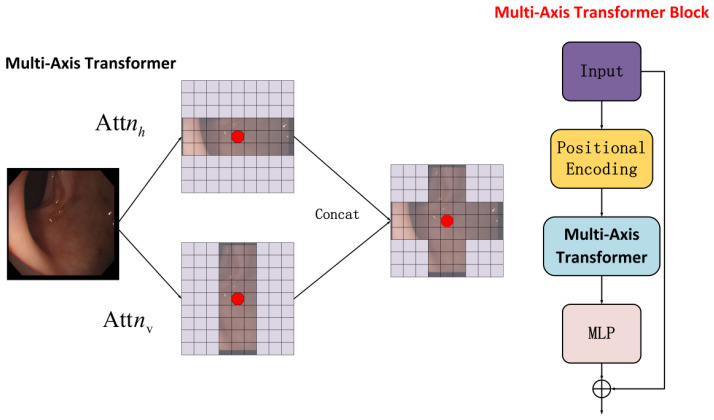
Architecture of MATransformerBlock. The block uses position encoding, horizontal and vertical attention branches, projection, MLP refinement, normalization, and residual fusion.

The main idea is to factorize global attention into horizontal and vertical branches. Compared with standard self-attention with complexity $O({\left(H\times W\right)}^{2}C)$, the proposed multi-axis approximation has complexity O(H×W×(H+W)C), which is more suitable for dense segmentation feature maps. The overall architecture of MATransformerBlock is illustrated in Figure 2.

First, a learnable 2D depthwise separable convolutional position encoding is applied to the input feature map $X\in R^{B\times C\times H\times W}$. Then, the features are processed by 1×1 convolutions. This process embeds spatial position information to address the Transformer’s insensitivity to absolute position:

(6) $$X_{\mathrm{pos}}=X+\mathrm{PosEnc}\left(X\right).$$

Subsequently, $X_{\mathrm{pos}}$ is reshaped into the sequence $S=\mathrm{Reshape}(X_{\mathrm{pos}})$ and the query, key, and value are generated by linear mapping:

(7) $$\left[Q,K,V\right]=\mathrm{Linear}(\mathrm{Reshape}\left(X_{\mathrm{pos}}\right)).$$

Then the horizontal and vertical dependencies of the image are captured separately, and *Q*, *K*, *V* are reshaped into horizontal branches $Q_{h}$, $K_{h}$, $V_{h}$ and vertical branches $Q_{v}$, $K_{v}$, $V_{v}$:

(8) $$Q=\mathrm{concat}(Q_{h},Q_{v}),$$

(9) $$K=\mathrm{concat}(K_{h},K_{v}),$$

(10) $$V=\mathrm{concat}(V_{h},V_{v}),$$

where $d_{k}$ is the dimension of key, $Q_{h}$, $K_{h}$, $V_{h}$ denote the query, key, and value in the horizontal direction, respectively, and $Q_{v}$, $K_{v}$, $V_{v}$ denote the query, key, and value in the vertical direction, respectively. This allows the model to effectively capture horizontal and vertical dependencies. By computing the horizontal and vertical self-attention results and fusing them, we obtain the cross-attention output:

(11) $${\mathrm{Attn}}_{h}=\mathrm{softmax}\left(\frac{Q_{h}K_{h}^{T}}{\sqrt{d_{k}}}\right)V_{h},$$

(12) $${\mathrm{Attn}}_{v}=\mathrm{softmax}\left(\frac{Q_{v}K_{v}^{T}}{\sqrt{d_{k}}}\right)V_{v},$$

(13) $${\mathrm{Attn}}_{\mathrm{out}}={\mathrm{Attn}}_{h}+{\mathrm{Attn}}_{v},$$

Next, ${\mathrm{Attn}}_{\mathrm{out}}$ is projected by a linear transformation to obtain $\mathrm{Proj}({\mathrm{Attn}}_{\mathrm{out}})$. At this point, the features are divided into two branches: one is the input feature *X* plus the position encoding; the other is the cross-attention projection output. The input feature *X* is then processed by the normalization and MLP modules and fused with the cross-attention output:

(14) $$Y_{\mathrm{fusion}}=\mathrm{MLP}\left(\mathrm{LN}\left(X\right)\right)+\mathrm{Proj}\left({\mathrm{Attn}}_{\mathrm{out}}\right),$$

Finally, the final output is obtained by passing it through LayerNorm one more time and concatenating it residually with the initial position-encoded input:

(15) $$Y_{\mathrm{final}}=\mathrm{LN}\left(Y_{\mathrm{fusion}}+X\right).$$

This design reduces computational complexity by using horizontal and vertical attention mechanisms with residual feature preservation. By decomposing global dependency modeling into two direction-wise processes, the module can capture elongated or irregular polyp structures along different axes while retaining the local representation learned by convolutional layers.

### 3.2. Cross-Channel Mixing (CCM) Module

In nested U-Net++ skip paths, direct concatenation can transmit redundant low-level texture responses and semantically inconsistent features to the decoder. The key limitation addressed here is therefore the lack of adaptive selection during multi-level feature fusion.

**Figure 3:**
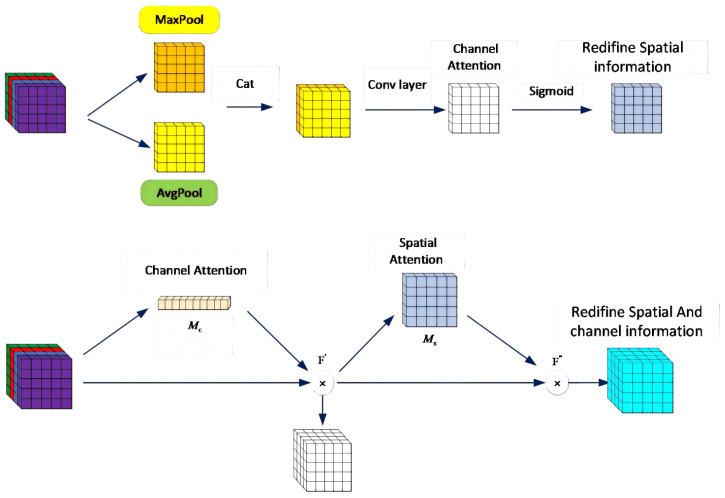
Cross-Channel Mixing module with channel attention and spatial attention operations.

From an information bottleneck viewpoint, the encoder features contain both task-relevant information and noisy details. CCM is therefore designed as an adaptive feature selector that attenuates redundant channels and spatial locations while amplifying semantically useful responses. As shown in Figure 3, CCM first applies global average pooling and max pooling in parallel to the input feature map, *F*, thereby capturing complementary global context information for subsequent fusion.

Let $F\in R^{B\times C\times H\times W}$ denote the input feature map, where H×W has the same meaning as defined above, namely the spatial resolution of the current feature level. To extract global channel statistics, the CCM module applies global average pooling (AvgPool) and max pooling (MaxPool):

(16) $$\mathrm{AvgPool}\left(F\right)=\frac{1}{HW}\sum_{i=1}^{H}\sum_{j=1}^{W}F_{i,j},$$

(17) $$\mathrm{MaxPool}\left(F\right)=\max_{i,j}F_{i,j}.$$

$F_{i,j}$ denotes the activation value at position (i,j) in the feature map. H×W denotes the spatial resolution of the feature map. $\max_{i,j}F_{i,j}$ denotes the maximum value obtained by traversing all spatial positions in the feature map. These pooling operations provide global statistical information about the input feature map at different scales, offering multi-scale support for subsequent feature fusion.

After pooling, the multi-scale feature representations are processed through a multilayer perceptron (MLP). In the MLP, features from each scale are assigned adaptive weights and fused. A channel attention mechanism is applied to adaptively weight channel-wise features. Specifically, the pooled features are projected through the MLP to obtain scale-specific weight vectors, which are then normalized using a sigmoid function to produce adaptive channel-wise attention maps for subsequent feature fusion:

(18) $$F_{\mathrm{channel}}=\mathrm{MLP}\left(\mathrm{concat}\left[\mathrm{AvgPool}\left(F\right),\mathrm{MaxPool}\left(F\right)\right]\right),$$

The Concat operation joins the pooled features from different scales, and the weighted information of the fused channels is obtained through MLP processing. Next, the Cross-Channel Mixing (CCM) module applies channel and spatial attention mechanisms to weight the features. The sigmoid function maps the weights to the [0,1] interval, reflecting the activation strength of each channel. Channel attention assigns adaptive weights to features across channels, and the final channel-weighted output is calculated as follows:

(19) $$F_{\mathrm{out}}^{\mathrm{chan}}=F⊙\sigma \left(F_{\mathrm{channel}}\right),$$

To further enhance spatial information attention, the CCM module introduces a spatial attention mechanism. This mechanism assigns different weights to various spatial locations by combining pooling results along the channel dimension and generating a spatial attention map through a convolution operation. The computation process of the spatial attention map is as follows:

(20) $$A_{\mathrm{spatial}}=\sigma \;\left(\mathrm{Conv}2d(\mathrm{concat}[\mathrm{MaxPool}(F),\mathrm{AvgPool}(F)])\right),$$

A 2D convolutional layer performs the convolution over the combined feature information to generate the spatial attention map. This allows the model to dynamically adjust its attention across image regions, improving segmentation accuracy.

After generating the weighted feature map through the channel and spatial attention mechanisms, the CCM module combines the weighted features with the input feature map. In our residual connection design, the final output feature is composed of the weighted sum of the original input feature and the attention-weighted feature:

(21) $$F_{\mathrm{out}}=F+\gamma \left(F_{\mathrm{out}}^{\mathrm{chan}}⊙A_{\mathrm{spatial}}\right).$$

In the residual connection design, *F* represents the original input features passed from the encoder by skipping connections, where $F_{\mathrm{out}}^{\mathrm{chan}}$ is the channel-weighted features and $A_{\mathrm{spatial}}$ is the spatial attention map. γ is a learnable parameter with a small initial value (such as 0.1), which ensures that, even if the attention mechanism is not well learned, the original feature can still be passed. This process accurately models important regions and details in the image while fusing multi-scale information.

By employing dual-attention gating mechanisms, CCM implements content-aware feature routing. Thus, multi-scale features are fused not only according to spatial alignment but also according to their contribution to the final segmentation task.

### 3.3. Multi-Objective Adaptive Loss (MOALoss)

In polyp segmentation, the foreground occupies only a small part of the image and lesion boundaries are often ambiguous. MOALoss is therefore designed to optimize three complementary objectives: focal supervision for hard pixels, Dice supervision for region overlap, and boundary-aware supervision for contour refinement.

Manually tuning the weights $\left[\lambda_{\mathrm{focal}},\lambda_{\mathrm{dice}},\lambda_{\mathrm{boundary}}\right]$ is unstable across datasets. Inspired by adaptive multi-task loss balancing [18], we use learnable weights to balance the three terms during training. The conceptual uncertainty-based formulation is written as

(22) $$L_{\mathrm{MOA}}=\frac{1}{2\sigma_{\mathrm{bce}}^{2}}L_{\mathrm{focal}}+\frac{1}{2\sigma_{\mathrm{dice}}^{2}}L_{\mathrm{dice}}+\frac{1}{2\sigma_{\mathrm{boundary}}^{2}}L_{\mathrm{boundary}}+\log\left(\sigma_{\mathrm{bce}}\sigma_{\mathrm{dice}}\sigma_{\mathrm{boundary}}\right),$$

Here, $\sigma_{\mathrm{bce}}$, $\sigma_{\mathrm{dice}}$, and $\sigma_{\mathrm{boundary}}$ are learnable parameters representing the intrinsic uncertainty of each task. The log term acts as a regularizer to prevent the uncertainties from becoming too large. In practice, to ensure numerical stability, we learn the parameters $s_{i}=\log\sigma_{i}^{2}$, and the loss becomes

(23) $$L_{\mathrm{MOA}}=\frac{1}{2}e^{-s_{\mathrm{bce}}}L_{\mathrm{focal}}+\frac{1}{2}e^{-s_{\mathrm{dice}}}L_{\mathrm{dice}}+\frac{1}{2}e^{-s_{\mathrm{boundary}}}L_{\mathrm{boundary}}+\frac{1}{2}\left(s_{\mathrm{bce}}+s_{\mathrm{dice}}+s_{\mathrm{boundary}}\right).$$

where $s_{\mathrm{bce}}$, $s_{\mathrm{dice}}$, and $s_{\mathrm{boundary}}$ are learnable parameters that correspond to the weights of each loss term.

In the implemented model, this adaptive idea is parameterized as learnable sigmoid-normalized weights initialized as $w_{\mathrm{focal}}=0.4$, $w_{\mathrm{dice}}=0.4$, and $w_{\mathrm{boundary}}=0.2$:

(24) $$L_{\mathrm{MOA}}=\sigma \left(w_{\mathrm{focal}}\right)L_{\mathrm{focal}}+\sigma \left(w_{\mathrm{dice}}\right)L_{\mathrm{dice}}+\sigma \left(w_{\mathrm{boundary}}\right)L_{\mathrm{boundary}}.$$

The implemented focal term uses focusing factor γ=2.0 without an additional class-balancing α coefficient, the Dice numerical stability parameter is $\epsilon =10^{-6}$, and the dynamic Dice smoothing additionally scales with the foreground area. The boundary map is computed by a signed Euclidean distance transform with a scaling factor of 0.1 and is used as a multiplicative weight in the boundary-aware binary cross-entropy term. When deep supervision is enabled, output-layer weights decay with δ=0.7; in the reported experiments, deep supervision was disabled.

**(1) Focal Cross-Entropy Loss:** In medical images, severe imbalance often exists between foreground and background pixels. Traditional Binary Cross-Entropy Loss (BCE) does not effectively address this. To mitigate this, the implemented focal term increases the model’s focus on hard-to-classify pixels through the focusing factor γ, without adding a separate class-balancing α coefficient. It is formulated as

(25) $$L_{\mathrm{focal}}=\mathrm{BCE}\left(z,y\right){\left(1-\exp[-\mathrm{BCE}(z,y)]\right)}^{\gamma },$$

where *z* denotes the predicted logit, *y* denotes the ground-truth label, and BCE(z,y) denotes binary cross-entropy with logits. A larger γ places more emphasis on difficult samples and reduces the contribution from easy-to-classify samples. This enables the focal term to strengthen learning in challenging regions, such as polyp edges, and reduce background interference.

**(2) Dice Loss:** The Dice coefficient is a common measure of overlap between predicted and ground-truth masks in medical image segmentation. To address class imbalance, we design adaptive Dice Loss to balance the contributions of foreground and background by introducing dynamic smoothing coefficients and weighting difficult samples. The formula is:

(26) $$L_{\mathrm{dice}}=1-\frac{2\sum_{i}y_{i}{\hat{y}}_{i}+\epsilon }{\sum_{i}y_{i}+\sum_{i}{\hat{y}}_{i}+\epsilon },$$

where *Y* is the true label (ground truth), $\hat{Y}$ is the probability map predicted by the model, and ∑ is the summation over all pixels (or regions) of the image. To further improve the model’s sensitivity to difficult regions, we introduce an adaptive weighting mechanism that adjusts each sample’s weight based on the discrepancy between *Y* and $\hat{Y}$. This enables the model to focus automatically on challenging segmentation regions, particularly small targets such as polyps.

**(3) Boundary perception loss:** Boundary perceptual loss is computed by distance transformation to compute boundary weights. Suppose the binary mask of the target region is *Y* and $\hat{Y}$ is the probability map predicted by the model. The distance transform is computed by the following formula to generate the Euclidean distance from each pixel to the nearest boundary:

(27) boundary_map=distance_transform(Y),

(28) $$L_{\mathrm{boundary}}=\mathrm{BCE}\left(Y,\hat{Y}\right)⊙\left(1+|\mathrm{boundary}\_\mathrm{map}|\right).$$

where standard Binary Cross-Entropy Loss, |boundary_map|, is the absolute value processing of distance transformation results. This way, the boundary region receives a higher weight and the model focuses more on accurately segmenting the target’s boundary during training, which is especially useful when dealing with small targets, such as polyps. This effectively reduces errors caused by boundary blurring.

Finally, we formulate the MOALoss function as a weighted combination of Focal Cross-Entropy Loss, Adaptive Dice Loss, and Boundary-Aware Loss, as defined in Equation (24).

## 4. Results and Discussion

This section reports comparative results, qualitative visualizations, theoretical analysis, ablation experiments, and a discussion of limitations. Dataset descriptions, metrics, and implementation details are provided in Section 2.

### 4.1. Comparative Experiments

To evaluate the effectiveness of the proposed method, we compared MCM-UNet++ with representative segmentation baselines, including U-Net, U-Net++, PraNet, SFA, and CaraNet [5,19,20,21], on Kvasir-SEG, CVC-ClinicDB, CVC-ColonDB, and ETIS-Larib. These baselines were selected because they cover classical encoder–decoder segmentation, nested skip connection segmentation, reverse-attention polyp segmentation, selective feature aggregation, and context-aware polyp segmentation. The same Dice and IoU metrics defined in Section 2.3 were used.

For fairness and reproducibility, the reported values are compared under the same metric definitions and fixed validation protocol used in this study. When a baseline result is reproduced locally, the same preprocessing and validation split are used; when an original-publication value is used as a reference, the method is cited accordingly.

Table 4 shows that MCM-UNet++ obtains the highest Dice and IoU values among the selected methods on Kvasir-SEG, CVC-ColonDB, and ETIS-Larib, and remains competitive on CVC-ClinicDB. For example, it achieves Dice/IoU values of 0.9563/0.9278 on Kvasir-SEG and 0.8593/0.7896 on CVC-ColonDB. The improvement over U-Net++ on CVC-ColonDB is consistent with the design motivation: global context modeling and adaptive skip fusion are helpful for irregular polyps and complex backgrounds.

Compared with PraNet, SFA, and CaraNet, MCM-UNet++ benefits from combining nested U-Net++ feature reuse with CCM and MATransformerBlock. Nevertheless, these comparisons should be interpreted as evidence of strong benchmark performance under the selected experimental setting rather than as a definitive claim of universal superiority.

To complement Dice and IoU, Table 5 reports additional image-level thresholded-mask metrics computed from the retained foreground prediction masks. These metrics provide additional information about false positives, false negatives, background rejection, probability error, and boundary localization.

We further performed a paired Wilcoxon signed-rank test using image-level Dice and IoU values where paired foreground prediction masks were available. The test compared MCM-UNet++ with the nested U-Net++ variant with deep supervision on the CVC-ClinicDB validation split. A one-sided alternative hypothesis was used to test whether MCM-UNet++ achieved higher image-level scores. As shown in Table 6, the improvements were statistically significant for both Dice and IoU.

**Figure 4:**
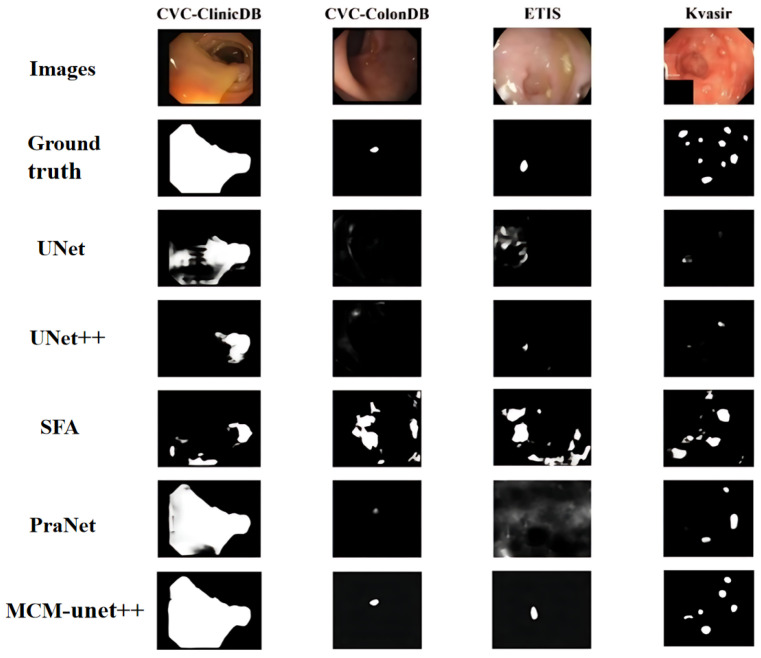
Visual comparison of segmentation results. The rows correspond to the original image, ground truth, U-Net, U-Net++, SFA, PraNet, CaraNet, and MCM-UNet++, respectively.

Figure 4 shows the segmentation results for the same endoscopic images across four datasets using six methods. The last row is labeled MCM-UNet++. Qualitatively, MCM-UNet++ delineates the polyp region more completely in challenging cases, whereas some competing methods produce missing regions or false positives in backgrounds with mucus texture and intestinal folds.

### 4.2. Theoretical Analysis

This subsection is provided as theoretical justification rather than as an additional comparative experiment. It explains the computational and representational motivation of the proposed modules.

#### 4.2.1. Complexity and Effective Receptive Field Analysis

We compare the theoretical computational complexity and parameter count of key components in Table 7. MATransformerBlock provides a trade-off between global contextual modeling and computational cost: it retains a global or near-global effective receptive field while avoiding the dense token interaction of standard self-attention.

#### 4.2.2. Visualizing the Role of CCM

**Figure 5:**
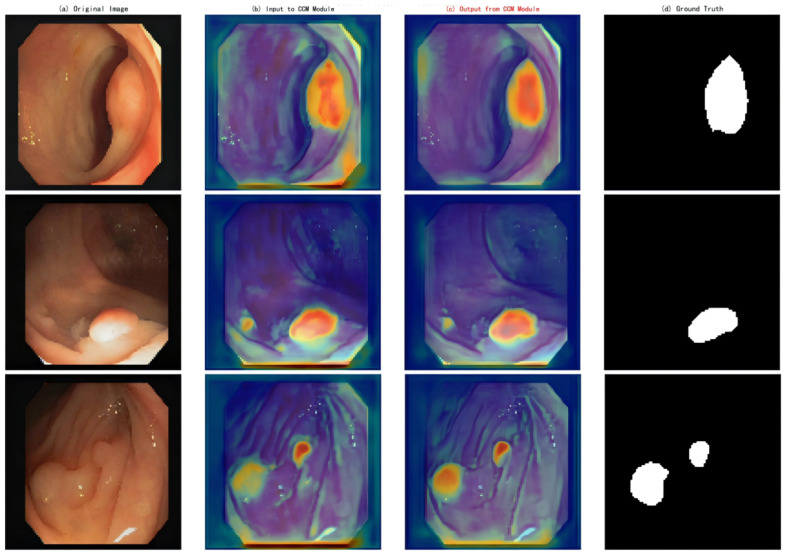
Feature filtering visualization before and after CCM, comparing input features and CCM-refined activations.

To qualitatively demonstrate the CCM module’s function as an information filter, we visualize the feature maps before and after CCM processing in Figure 5. Before CCM, activations are distributed over both polyp and background regions. After channel and spatial attention, the responses become more concentrated around the polyp and its boundary. This supports the design hypothesis that CCM suppresses redundant skip features and helps the decoder focus on task-critical regions.

#### 4.2.3. Implication of Adaptive Loss Balancing

The learnable weights in MOALoss provide insight into the optimization process. Boundary-related weights receive larger effective contributions when the masks contain blurred contours, indicating that adaptive loss balancing can emphasize boundary refinement without manually tuning a fixed coefficient for every dataset.

### 4.3. Ablation Experiments

To assess the independent contribution of each module, we introduced CCM, MATransformerBlock, and MOALoss into the U-Net++ baseline and evaluated the resulting variants. The ablation study was conducted on the CVC-ColonDB validation split described in Table 1; therefore, the U-Net++ baseline in Table 8 is consistent with the CVC-ColonDB result in Table 4.

The table shows that all three modules contribute to performance improvements:

- **CCM module:** Integrating only CCM increased Dice from 0.4830 to 0.8012 and IoU from 0.4100 to 0.7307, indicating that adaptive nested skip fusion strongly improves multi-scale feature interaction.
- **MATransformerBlock:** Adding only MATransformerBlock improved Dice to 0.7822 and IoU to 0.7041, validating the benefit of long-range context modeling for irregular polyp boundaries.
- **MOALoss:** Replacing BCE+Dice with MOALoss increased Dice to 0.8172 and IoU to 0.7534, showing that adaptive multi-objective supervision helps balance foreground, overlap, and boundary learning.
- **Full-module integration:** Combining CCM, MATransformerBlock, and MOALoss achieved the best performance, with Dice of 0.8593 and IoU of 0.7896, suggesting that the modules provide complementary gains.

Additional ablations for different loss combinations, the number and location of MATransformerBlocks, and higher input resolutions will further strengthen this analysis and are identified as future work.

### 4.4. Discussion, Limitations, and Future Work

The experimental results suggest that the proposed components address different aspects of the polyp segmentation problem. MATransformerBlock improves long-range contextual modeling, which is useful for irregularly shaped lesions. CCM reduces redundant skip-path information and makes decoder features more focused on lesion regions. MOALoss complements these architectural changes by emphasizing hard foreground pixels, region overlap, and boundary-sensitive errors. The ablation results in Table 8 support the complementary roles of these modules.

Several limitations remain. First, the reported results are based on fixed public dataset splits, and no external multi-center clinical validation was conducted. Second, the input sizes used in the reported runs (96×96 for three datasets and 128×128 for ETIS-Larib) were chosen for computational feasibility on the available GPU, but downsampling may lead to the loss of fine boundary details. Third, the reported results are fixed-seed results rather than mean and standard deviation over multiple independent runs. Fourth, although the selected baselines cover representative U-Net-, attention-, and polyp-specific segmentation models, broader comparison with more recent architectures would further strengthen the empirical evidence.

Future work will therefore investigate higher-resolution training, repeated-run statistical reporting, additional recent baselines, external validation on multi-center colonoscopy data, and lightweight deployment strategies. Extending the method to video-level segmentation is also promising because temporal consistency can help reduce frame-level false positives and boundary flicker.

## 5. Conclusions

This study addresses three common challenges in automatic polyp segmentation: limited global context, redundant skip-path fusion, and imbalanced boundary-sensitive supervision. To this end, we proposed MCM-UNet++, a U-Net++-based framework that incorporates MATransformerBlock for multi-axis context modeling, CCM for adaptive nested feature fusion, and MOALoss for multi-objective optimization. Experiments on Kvasir-SEG, CVC-ClinicDB, CVC-ColonDB, and ETIS-Larib show that the proposed model achieves competitive performance among the selected baselines, with particularly clear improvements on CVC-ColonDB and ETIS-Larib. These findings indicate that combining efficient global context modeling, adaptive feature selection, and boundary-aware loss balancing is a useful direction for benchmark-level polyp segmentation. Broader baseline comparisons, higher-resolution experiments, repeated-run statistics, and external clinical validation are needed before making stronger claims about generalization or clinical deployment.

## Figures and Tables

**Table 1:** Dataset split protocol used in this study.

Dataset	Total	Training	Validation	Split Protocol
Kvasir-SEG	1000	800	200	Fixed 8:2 split, seed 41
CVC-ClinicDB	612	489	123	Fixed 8:2 split, seed 41
CVC-ColonDB	380	304	76	Fixed 8:2 split, seed 41
ETIS-Larib	196	156	40	Fixed 8:2 split, seed 41

**Table 2:** Implementation and training configuration.

Dataset	Input Size	Epochs	Batch Size	Momentum
Kvasir-SEG	96×96	100	8	0.900
CVC-ClinicDB	96×96	200	8	0.937
CVC-ColonDB	96×96	100	4	0.900
ETIS-Larib	128×128	200	4	0.937

**Table 3:** Main architectural configuration of MCM-UNet++.

Stage	Spatial Size	Channels	Heads	Configuration
Encoder level 0	96×96	32	4	Two 3×3 conv. layers plus MATransformerBlock
Encoder level 1	48×48	64	4	Two 3×3 conv. layers plus MATransformerBlock
Encoder level 2	24×24	128	4	Two 3×3 conv. layers plus MATransformerBlock
Encoder level 3	12×12	256	4	Two 3×3 conv. layers plus MATransformerBlock
Encoder bottleneck	6×6	512	4	Two 3×3 conv. layers plus MATransformerBlock
Nested decoder nodes	Multi-scale	32–256	4	CCM, convolutional refinement, and MATransformerBlock
Prediction head	96×96	1	–	1×1 convolution for binary segmentation

**Table 4:** Comparative segmentation results on four public polyp datasets.

Method	Kvasir-SEG		CVC-ClinicDB		CVC-ColonDB		ETIS-Larib	
	Dice	IoU	Dice	IoU	Dice	IoU	Dice	IoU
U-Net	0.7900	0.7300	0.7800	0.7500	0.4600	0.3300	0.3980	0.3250
U-Net++ [5]	0.8210	0.7430	0.7940	0.7290	0.4830	0.4100	0.4010	0.3440
PraNet [19]	0.8980	0.8400	0.8990	0.8490	0.7090	0.6400	0.6280	0.5670
SFA [20]	0.7230	0.6110	0.7000	0.6070	0.4690	0.3470	0.2970	0.2170
CaraNet [21]	0.9180	0.8650	0.9360	0.8870	0.7730	0.6890	0.7470	0.6720
**MCM-UNet++**	**0.9563**	**0.9278**	**0.9191**	**0.8616**	**0.8593**	**0.7896**	**0.8334**	**0.7407**

*Note*: Bold values indicate the best performance in each column.

**Table 5:** Extended image-level evaluation of MCM-UNet++ on validation splits with retained foreground prediction masks.

Dataset	Precision	Recall	Specificity	Accuracy	MAE	HD95
CVC-ClinicDB	0.8947	0.9045	0.9923	0.9854	0.0158	5.2387
CVC-ColonDB	0.8422	0.8202	0.9896	0.9808	0.0233	5.4527
ETIS-Larib	0.7711	0.7212	0.9930	0.9857	0.0143	16.8364

**Table 6:** Paired Wilcoxon signed-rank test using image-level validation scores.

Dataset	Comparison	*n*	Dice Mean Difference	Dice *p*-Value	IoU *p*-Value
CVC-ClinicDB	MCM-UNet++ vs. Nested U-Net++ (DS)	123	0.0231	$5.32\times 10^{-8}$	$2.04\times 10^{-8}$

**Table 7:** Theoretical comparison of key components.

Component	Computational Complexity	Parameters	Effective Receptive Field
3 × 3 Convolution	$O(HWC^{2}\cdot 9)$	$9C^{2}$	Local
Standard Self-Attention	$O({\left(HW\right)}^{2}C)$	$3C^{2}$	Global
MATransformerBlock (ours)	O(HW(H+W)C)	$3C^{2}+k$	Global (structured)
CCM Module (Ours)	O(HWC)	Negligible	–

**Table 8:** Ablation results on the CVC-ColonDB validation split.

U-Net++	CCM	MATransformer Block	MOALoss	Dice	IoU
✓				0.4830	0.4100
✓	✓			0.8012	0.7307
✓		✓		0.7822	0.7041
✓			✓	0.8172	0.7534
✓	✓	✓	✓	**0.8593**	**0.7896**

*Note*: ✓ indicates that the corresponding module is included, and bold values indicate the best performance.

## Data Availability

The data used in this study are publicly available. Kvasir-SEG can be accessed via the following link: https://datasets.simula.no/kvasir-seg/ (accessed on 18 May 2026); CVC-ClinicDB can be downloaded from https://polyp.grand-challenge.org/CVCClinicDB/ (accessed on 18 May 2026); CVC-ColonDB can be obtained from https://www.kaggle.com/datasets/longvil/cvc-colondb (accessed on 18 May 2026); ETIS-Larib is available at https://zenodo.org/records/5579392 (accessed on 18 May 2026). All data generated or analyzed during this study are included in the published article. Access to the data may also be requested by contacting the author, Ming Zhao, at hitmzhao@gmail.com, due to privacy or ethical concerns.
